# The Impact of the Coronavirus Disease (COVID-19) on the Health and Social Needs of Sex Workers in Singapore

**DOI:** 10.1007/s10508-021-01951-8

**Published:** 2021-06-30

**Authors:** Rayner Kay Jin Tan, Vanessa Ho, Sherry Sherqueshaa, Wany Dee, Jane Mingjie Lim, Jamie Jay-May Lo, Alvin Kuo Jing Teo, Caitlin Alsandria O’Hara, Clarence Ong, Ann Hui Ching, Mee Lian Wong

**Affiliations:** 1 Saw Swee Hock School of Public Health, Tahir Foundation Building, National University of Singapore, 12 Science Drive 2, #10-01, Singapore, 117549 Singapore; 2 Project X, Singapore, Singapore; 3 Yong Loo Lin School of Medicine, National University of Singapore, Singapore, Singapore

**Keywords:** COVID-19, Sex workers, Stigma, Food insecurity, Housing insecurity

## Abstract

We evaluated the impact of the coronavirus disease (COVID-19) on the sex work industry and assessed how it has impacted the health and social conditions of sex workers in Singapore. We conducted a sequential exploratory mixed methods study amidst the COVID-19 pandemic from April to October 2020, including in-depth interviews with 24 stakeholders from the sex work industry and surveyor-administered structured surveys with 171 sex workers. COVID-19 had a substantial impact on sex workers' income. The illegality of sex work, stigma, and the lack of work documentation were cited as exclusionary factors for access to alternative jobs or government relief. Sex workers had experienced an increase in food insecurity (57.3%), housing insecurity (32.8%), and sexual compromise (8.2%), as well as a decrease in access to medical services (16.4%). Being transgender female was positively associated with increased food insecurity (aPR = 1.23, 95% CI [1.08, 1.41]), housing insecurity (aPR = 1.28, 95% CI [1.03, 1.60]), and decreased access to medical services (aPR = 1.74, 95% CI [1.23, 2.46]); being a venue-based sex worker was positively associated with increased food insecurity (aPR = 1.46, 95% CI [1.00, 2.13]), and being a non-Singaporean citizen or permanent resident was positively associated with increased housing insecurity (aPR = 2.59, 95% CI [1.73, 3.85]). Our findings suggest that COVID-19 has led to a loss of income for sex workers, greater food and housing insecurity, increased sexual compromise, and reduced access to medical services for sex workers. A lack of access to government relief among sex workers exacerbated such conditions. Efforts to address such population health inequities should be implemented.

**Supplementary Information:**

The online version contains supplementary material available at 10.1007/s10508-021-01951-8.

## Introduction

The coronavirus disease 2019 (COVID-19) pandemic was declared by the World Health Organization as a public health emergency of international concern on January 30, 2020, and has since severely impacted the livelihood of individuals around the world (World Health Organization, 2020). Inevitably, this impact has been felt disproportionately among vulnerable populations that face substantial structural disparities in access to medical resources and are predisposed to poorer health and socioeconomic conditions (Raifman & Raifman, 2020). This includes sex workers, a key vulnerable population at heightened risk of sexual violence, food insecurity, homelessness, as well as acquiring HIV and other sexually transmitted infections (STI) (Collison & Christianson, 2020; France-Presse, 2020; Global Network of Sex Work Projects, 2020b; Joseph, 2020).

Data were rapidly emerging that the COVID-19 pandemic was exacerbating the hardships that sex workers face around the world (UNAIDS, 2020b). Specifically, sex workers have experienced a loss of clients and income as a result of the measures to curb the spread of COVID-19 (UNAIDS, 2020a). Not only does the loss of income faced by sex workers make them more financially vulnerable, but they are further placed in a compromised position whereby clients push boundaries and sex workers face increased pressure to take risks while working in order to compensate for their loss of income (Donoho, 2020; Nielsen, 2020; Star, 2020). While the loss of clients has led to some sex workers transitioning to online work where physical interactions with clients are not necessary, there are many barriers to entry for online work—not all sex workers may be able to sustain their livelihood through such means (Voynovskaya, 2020).

As a key population already vulnerable to the acquisition of HIV and other STIs, disruptions in accessing care and medications for STI prevention and treatment due to COVID-19 control measures have exacerbated their vulnerabilities (Global Network of Sex Work Projects, 2020b). This is further compounded for sex workers who operate in settings where punitive measures linked to sex work, HIV, and COVID-19-related laws prevail. Increased policing can expose sex workers to more harassment and violence—home raids, compulsory COVID-19 testing, arrest, and threatened deportation of migrant sex workers (Global Network of Sex Work Projects, 2020b). Furthermore, sex workers are often left out of government-level economic support or relief packages meant for registered freelance workers to mitigate the impact of COVID-19 on their livelihood. While some countries have offered street-based sex workers relief, sex workers have noted that these payouts have not been enough for them due to a lack of food and shelter security (Global Network of Sex Work Projects, 2020a).

While sex work remains illegal in Singapore, there exist government-regulated and licensed brothels where sex work can take place by registered, typically non-local, sex workers. Part of such regulation involves the coverage of these brothels under the medical surveillance scheme, where registered sex workers are required to attend the only public STI clinic and designated general practice clinics in Singapore for regular screening for sexually transmitted infections (Sen, Chio, Tan, & Chan, 2006). Singapore has recently witnessed a major shift in sex work from such regulated, licensed brothels, toward online and street-based settings as well as massage parlors, beauty salons, and entertainment establishments such as karaoke lounges, night clubs and discotheques (Lim et al., 2018). As such latter forms of sex work remain illegal and unregulated, sex workers operating in such settings may experience a lack of access to social and health services. There are an estimated 10,000 sex workers in Singapore, with less than 1000 (10%) registered under the legal medical surveillance scheme (Teo et al., 2019).

Given that sex workers are prone to be displaced to hidden settings due to sex work, HIV, and COVID-19-related laws and may experience greater disparities in health outcomes, further research into their situation as a result of the COVID-19 pandemic is warranted. Specifically, this study employs a sequential mixed methods study design to investigate how COVID-19 and its concomitant movement control measures have directly impacted the livelihood of sex workers, which was a broad research question that our team undertook as we initiated this study. As our study proceeded, themes that were generated from our initial qualitative phase were subsequently investigated through a quantitative survey questionnaire.

## Method

### Study Design

We conducted a sequential exploratory mixed methods study comprising in-depth interviews with 24 stakeholders in the sex work industry and subsequently 171 surveyor-administered surveys with sex workers in Singapore. A sequential exploratory mixed methods study typically combines both an exploratory qualitative study, followed by a quantitative study in a sequence of phases (Creswell & Plano Clark, 2018). Singapore’s lockdown, also known as the “circuit breaker,” involved the closure of all but essential workplaces from April 7 until June 1, 2020, alongside strictly enforced movement control measures during which our stakeholder interviews were conducted. This also involved the closure of regulated, licensed brothels and other venues where sex work may take place, such as beauty salons, massage parlors, clubs and bars, and other entertainment establishments. The term “circuit breaker” refers to this set of measures that would curb the continued spread of COVID-19 in the community, and in effect “break the circuit” of transmission (Government of Singapore, 2020). Movement control measures during this time included the closure of entertainment establishments and non-essential workplaces, mandatory mask-wearing, as well as restrictions on leaving the house unless for essential services such as buying groceries, caring for elderly family members who were living alone, or seeking or rendering medical attention. Stakeholders for the first phase of the study were selected to provide a holistic account from the perspectives of clients of sex workers, sex workers themselves, organizations whose beneficiaries comprise sex workers, as well as academics and policymakers who have conducted research on the sex work industry in Singapore.

The circuit breaker measures were then gradually eased in phases from June 2, 2020, during which our surveys with sex workers in the field were conducted. We were able to access sex workers in the field during this time as sex workers operating out of beauty salons, massage parlors, and other venues were allowed to reopen their respective premises. The in-depth interviews allowed the team to qualitatively explore and generate themes around how COVID-19 had impacted sex workers in Singapore, and informed the design of the quantitative surveyor-administered survey questionnaire. Overall, our qualitative phase allowed us to generate a series of themes and codes, and the quantitative survey allowed us to quantify the extent to which sex workers had experienced hardships in several areas. The present article considers the available data generated from both phases and synthesizes key qualitative themes and quantitative results into three broad themes that focus on the impact of the COVID-19 pandemic on the health and social needs of sex workers in Singapore. These three broad themes have been reported as subsections in our results. Ethics approval was granted by the Saw Swee Hock School of Public Health Department Ethics Review Committee, National University of Singapore (Reference number: SSHSPH-028) and the National University of Singapore Institutional Review Board (Reference number: S-20-136).

### Qualitative Phase: Participants, Data Generation, and Analysis

Stakeholders had to be at least 21 years old to be eligible to participate in the interviews. Interviewees were purposively sampled and recruited for this study through recommendations by Project X (https://theprojectx.org/), a community-based organization (CBO). Project X is a key CBO that addresses the health, social needs, and well-being of sex workers in Singapore. Table 1 summarizes the characteristics of participants by participant identification numbers, organization type, expert perspective, as well as the roles of the participants. Organizations' names have been removed to protect the identity of participants in this study.

**Table 1:** Stakeholder characteristics for in-depth interviews

Perspectives	Type	Role	ID
Clients of sex workers	Community-based organization	Program leader	01
Clients of sex workers	Community-based organization	Program executive	02
Sex workers and clients of sex workers	Community-based organization	Executive	05
Sex workers and clients of sex workers	Academia	Researcher	07
Sex workers and clients of sex workers	Academia	Researcher	10
Sex workers and clients of sex workers	Commercial entity	Manager	13
Sex workers and clients of sex workers	Government-related entity	Physician	16
Sex workers and clients of sex workers	Government-related entity	Policymaker	17
Sex workers and clients of sex workers	Government-related entity	Program Leader	22
Sex workers and transgender people	Community-based organization	Program leader	03
Sex workers	Community-based organization	Program manager	04
Sex workers	Community-based organization	Coordinator	06
Sex workers	Community-based organization	Peer volunteer	08
Sex workers	Community-based organization	Program leader	09
Sex workers	Community-based organization	Peer volunteer	11
Sex workers	Community-based organization	Peer volunteer	12
Sex workers	Community-based organization	Peer volunteer	14
Sex workers	Government-related entity	Program leader	15
Sex workers	Community-based organization	Peer volunteer	18
Sex workers	Community-based organization	Program Manager	19
Sex workers	Community-based organization	Coordinator	20
Sex workers	Community-based organization	Director	21
Sex workers	Commercial entity	Staff	23
Sex workers	Commercial entity	Owner	24

As strict movement control measures were implemented during this study, all interviews were conducted over phone calls or an online teleconferencing application or software (examples include Zoom). Participants were provided an electronic copy of the participant information sheet prior to the interview, and participants gave verbal consent to participate in the study before commencing the interview. A topic guide developed in consultation with academic staff and community leaders was used to guide the flow of the interviews. Questions were posted on the interviewee's organization, perspectives on sex work in Singapore, the impact of COVID-19 on the sex work industry and its stakeholders, and recommendations for policymakers to address the vulnerabilities of sex workers and the sex work industry from the impact of COVID-19. A copy of the semi-structured interview guide has been included as Supplemental Material. Interviews were conducted by trained qualitative researchers in the research team who have experience with interviewing vulnerable groups such as sex workers and patients living with stigmatized diseases (JML, JJL, RKJT, AKJT, WML). Interviews were conducted in both English and Chinese languages and were between 30 and 60 min each. All interviews were audio-recorded and subsequently transcribed verbatim. All interviews that were conducted in Chinese were transcribed verbatim and subsequently translated to English by a native Chinese speaker. These interviews were also quality-checked for completeness of the transcription and accuracy of translated data by another native Chinese speaker prior to analysis, following which no issues were raised and all transcripts were accepted for use in analysis. Participants were provided reimbursement of a Singapore dollar (SGD) 50.00 shopping voucher for their participation in the interview.

We adopted the framework method of qualitative data analysis to generate a coding frame and subsequently applied the agreed-upon framework to the data (Gale, Heath, Cameron, Rashid, & Redwood, 2013). The framework method, as argued by Gale et al. (2013), has “no allegiance to either inductive or deductive thematic analysis; where the research sits along this inductive-deductive continuum depends on the research question.” We did not utilize any pre-determined theory or framework, and thus, the analytic process involved transcribing and familiarizing ourselves with the data, independent coding of transcripts by members of the research team, developing a working analytical framework from the initial codes which was subsequently applied to the data, further revisions of the framework through subsequent coding, and finally, interpreting the data for reporting. A copy of our coding frame has been included as Supplemental Material. This coding frame and eventual themes generated from analyses were used to inform the design of the survey questionnaire.

### Quantitative Phase: Participants, Data Collection, Variable Measures and Statistical Analysis

Participants for the surveyor-administered, structured surveys were recruited through Project X's network of sex worker beneficiaries in Singapore. Participants had to be at least 21 years of age and self-identified as sex workers to participate in this study. These participants were chosen to reflect various subgroups of sex workers stratified along the lines of gender identity, venues of work, as well as nationality, who had remained in Singapore throughout the lockdown period. This approach was decided on based on consultation with Project X, as well as a review of the literature on the types of sex workers in Singapore (Delbyck, Jordan-Davis, Kwon, & Thoreson, 2015; Lim et al., 2018). Specifically, the surveys attempted to capture the experiences of transgender and cisgender female sex workers and cisgender male sex workers, sex workers who operated out of brick-and-mortar venues and online, as well as both Singaporean and foreign sex workers. Participants were approached and recruited consecutively by surveyors either in-person or by phone call; in-person surveys were solicited by members of the research team who had accompanied the Project X's team on their outreach efforts to venue-based sex workers, while phone surveys were solicited through Project X's network of beneficiaries. A total of 116 (67.8%) participants were recruited through in-person surveys.

All surveys began with the surveyor reading out the participant information sheet to participants and subsequently obtaining verbal consent from participants to participate in the study. The surveys each took an average of 20 min to complete. A total of seven surveyors (RKJT, VH, SS, WD, JML, JJL, AKJT, CO) conducted fieldwork alongside Project X’s outreach team, and attended a training session prior to the data collection period to ensure that any uncertainties in the survey instrument were discussed and resolved. The surveys were developed and primarily conducted in the English language, while the team engaged volunteer translators from the sex work community to ensure that sex workers who were not fluent in English were able to comprehend the questions that were being asked fully. These volunteer translators were also trained prior to assist in the data collection process. Participants were provided reimbursement of SGD25.00 cash for their participation in the survey. As the team was not able to enumerate the number of sex workers that were present in a given venue, and as some venues had gatekeepers, the response rate of the survey could not be calculated. However, all sex workers who accepted the invitation to participate in the survey completed it.

The survey collected key sociodemographic variables from participants that were identified throughout the stakeholder interviews, which included age (continuous variable), gender identity (cisgender female, transgender female, and cisgender male), living arrangements (staying in or renting own home, staying with others or at work), channels for accessing sex work (venue-based [e.g., entertainment establishments or brothels], online or street-based sex work), as well as nationality (Singapore citizen, permanent resident, or foreigner). The survey also collected several outcome variables, including income (in SGD) before COVID-19 and after COVID-19, from which absolute and relative (in %) decreases in income were derived. Cisgender male participants were excluded from multivariable analyses due to small sample size. A copy of the survey questionnaire has been included as Supplemental Material.

Food insecurity was ascertained through responses to two statements (“I worried whether my food would run out before I have the money to buy more” and “The food I bought just didn't last and I didn't have money to get more”) to which participants could select *Often True, Sometimes True, Never True and Don’t Know*. Participants were asked to respond similarly for statements on sexual compromise (I had to compromise on my own usual sexual health practices with my clients), as well as access to medical care (I was able to access medical or healthcare services that I needed). Participants who selected *Often True* or *Sometimes True* to any of these questions were determined to have experienced such conditions. Housing insecurity was ascertained through responses to two statements (“Was there a time when you were not able to pay rent on time?” and “In terms of where you stay, have you had to move two times or more within a month?”) to which participants could select *Yes*, *No* and *Don’t Know*. Participants who selected *Yes* to either question were determined to have experienced housing insecurity. Participants were asked to respond to all of these questions for the 12 months prior to COVID-19, as well as at present for comparison, and we were thus able to report the extent of participants’ usual experiences, as well as changes to these experiences as a result of the COVID-19 pandemic. Compared to the period prior to COVID-19, a change from *No* to *Yes*, as well as from *Never true* to *Sometimes true* or *Often true*, or *Sometimes true* to *Often true* indicated an increase in such conditions, and vice-versa.

The study initially attempted to recruit 369 sex workers to achieve a 95% confidence level and a 5% margin of error, based on an estimated population size of 10,000 sex workers in Singapore (Teo et al., 2019). However, this proved difficult as most foreign sex workers were no longer in Singapore at this time. With the eventual sample size of 171, we were able to obtain results at a 95% confidence level and a 7.5% margin of error. We employed descriptive statistics to summarize the trends in responses provided by respondents and also utilized multivariable linear and Poisson regression analyses with robust sandwich variances (Tai, 2013a, b), adjusting for key demographic variables of age, gender, living arrangements, sex work venues, and nationality to assess relationships between these key demographics and outcome variables in this study, including measures of food insecurity, housing insecurity, sexual compromise, as well as access to medical services. Statistical significance was set at *p* < .05 for the study.

## Results

### Impact of COVID-19 on Sex Workers' Health and Social Conditions

A summary of our qualitative themes and corresponding quotes is found in Table 2, and additional illustrative quotes that stem from these themes were included in the prose below. Stakeholders interviewed in the qualitative component of the study identified how the COVID-19 pandemic and its concomitant movement control measures had led to a loss of jobs due to the closure of venues where sex work had typically taken place:

**Table 2:** Key themes, subthemes, and illustrative quotes generated from qualitative phase of study

Themes	Subthemes	Illustrative quotes
Impact of COVID-19 on sex work	Impact of venue closures	After the licensed brothels and entertainment establishments were told to shut down, a week later, the unlicensed brothels were told to shut down. Since then, it has been three weeks. Since then we don’t go to work and we don’t see an increase or decrease. (ID14)
	Impact of movement control measures	Much quieter, really much quieter. There is a lot of fear […] there is fear with so much restrictions, laws and fines nowadays. I think it has been much quieter, a lot of movement activities are really toned down, including the sex industry. Really quiet. (ID01) We don’t know what is happening because we still dare not step into our workplace because the police are very strict. If we get caught, we get fined. So we are very worried about that. So even though we are staying in this district, we cannot go to that district. We get fined. (ID12)
	Additional demands due to COVID-19: Food security, housing security, access to medical services, sexual compromise	So some of them have voiced their worries, their concerns that they couldn’t afford to pay their rent. (ID04) It was going to be a very tough time, difficult time ahead for all of us. Because we really need to earn a ricebowl, make a living, to put food on our table. We were really apprehensive about it. (ID18) I have two cases of high blood pressure. And then, we had to get the drugs for them. I have got a volunteer to help run errands to bring one of the ladies to the doctor. […] The drugs weren’t easily available; so it is pretty chaotic like that. (ID04) That is an aspect I think many people overlook. We are not a very cushy kind of industry […] we do occasionally encounter drunk customers, customers who are tipsy and such is the consequence of that. We do come across not only verbal but also physical abuse. It is very real that physical assault may occur under such circumstances. (ID18)
Lack of COVID-19 relief and opportunities	Stigma of being a sex worker	As long as we disclose the nature of our professions, our work, it all might have a bearing on police investigation and how society in general look upon us. I feel. They feel, I think society does not think this is a legitimate kind of profession. (ID18)
	Stigma of being transgender	The sex industry exists because there is discrimination against transgender people in the workforce. Just because there is COVID doesn’t mean discrimination against them disappears. Asking them to find another job is like double whammy. (ID09)
	Lack of paperwork to qualify for relief	It’s gonna be hard because we don’t have any records. It may be hard for us to justify that they are self-employed. (ID13)
Adjustments in response to COVID-19	Carrying on sex work	I am sure it is happening. It already happened already. It is just that it is more underground, more secretive, come to the house must hide hide hide, or do in the car park or whatever. The man drive. Now cannot come out of the car. (ID03)
	Doing nothing	I have not been able to work. I don’t want to risk being fined or whatever, or to be caught loitering around or not running on essential errands. I don’t want to risk being fined. Have to wait till this period is over; hopefully, there will be no extension and we can resume our work safely and confidently without any fear. That is my hope. (ID18)
	Finding part-time / ad-hoc work	You know, there’s a [supermarket] opposite me? I asked for work and interviewed with them. But they said they are full. I had to fill up a form. If they have a vacancy at an alternative branch, they would give me a call. But until now, there has still been no vacancy. (ID23)

After the licensed brothels and entertainment establishments were told to shut down, a week later, the unlicensed brothels were told to shut down. Since then, it has been three weeks. Since then, we have not gone to work. (Peer volunteer, CBO)

Stakeholders also identified how sex workers could not go out to work out of fears of getting caught for breaching such movement control measures as a result of enhanced contact tracing. One manager at a commercial entity explained how sex workers, who were living in densely populated conditions, were worried about contracting COVID-19 and potentially infecting others living in the same house. The manager also briefly mentioned how the heightened risk of infection, and the prospect of enhanced contact tracing, might have criminal implications as well:

They [sex workers] are worried that if they were to go out and find other customers, or stay at others’ houses, if anything were to happen, it would be an even bigger problem for them to settle. […] If one person goes out, they come back with the virus—their rooms have 15, 16 people. (Manager, Commercial entity)

Stakeholders also articulated how the lack of work has led to sex workers in Singapore facing issues with paying rent on time, taking up loans, purchasing food, and sending money to family members whom they need to support:

Most of them [sex workers], they have voiced their concern and they have been wanting to go home since there is no work anyway. So there are overheads, they have to pay their loans. On top of that, they have to pay their rent. There is no income and they have to feed themselves. And they can’t send money home to their school-going children. So it is a very big burden. (Program Manager, CBO)

Our survey findings among sex workers also corroborated these perspectives—comparing between 12 months prior to COVID-19 and now, a total of 57.3% (*n* = 98), 32.8% (*n* = 56), 8.2% (*n* = 14) and 16.4% (*n* = 28) experienced increased food insecurity, increased housing insecurity, increased sexual compromise as well as decreased access to medical or healthcare services, respectively. Table 3 summarizes the sociodemographic characteristics of survey participants.

**Table 3:** Sociodemographic attributes and description of the analytic sample (n = 171)

Demographic variables	*n*	%	Mean	SD
Age (in years)			41.00	11.48
*Gender*				
Cisgender female	106	62.0		
Transgender female	60	35.1		
Cisgender male	5	2.9		
*Living arrangements*				
Stays in or rents their own home	121	70.8		
Stays with others or at work	50	29.2		
*Channels for accessing sex work*				
Venue-based sex work	126	73.7		
Online or street-based sex work	45	26.3		
*Nationality*				
Singapore citizen	102	59.7		
Singapore permanent resident	35	20.5		
Non-Singaporean	34	19.9		
Eligibility and application for Temporary Relief (n = 107)*				
Eligible and successfully applied	77	72.0		
Eligible but did not apply	12	11.2		
Not eligible	18	16.8		
*Food insecurity*				
Sometimes or often true before COVID-19	49	28.7		
Sometimes or often true after COVID-19	116	67.8		
Increase in food insecurity**	98	57.3		
*Housing insecurity*				
Sometimes or often true before COVID-19	41	24.0		
Sometimes or often true after COVID-19	85	49.7		
Increase in housing insecurity**	56	32.8		
*Sexual compromise*				
Sometimes or often true before COVID-19	25	14.6		
Sometimes or often true after COVID-19	28	16.4		
Increase in sexual compromise**	14	8.2		
*Good access to medical or healthcare services*				
Never true before COVID-19	45	26.3		
Never true after COVID-19	56	32.8		
Decrease in access to medical or healthcare services**	28	16.4		
*Income*				
Before COVID-19 (SGD)			3428.23	3808.65
After COVID-19 (SGD)			1119.70	1872.97
Absolute difference (SGD)			2291.29	2849.38
Relative difference (%)			67.76	33.22

SD, Standard Deviation; COVID-19, Coronavirus Disease 2019; SGD, Singapore Dollars

*Only Singapore citizens and permanent residents who were aware of such relief were asked (*n* = 107)

**Compared to the period prior to COVID-19, a change from “No” to “Yes”, as well as from “Never true” to “Sometimes true” or “Often true”, or “Sometimes true” to “Often true” indicated an increase in such conditions, and vice-versa

Another key area of concern, as a result of these worsening health and social conditions for sex workers, is the impact of psychosocial stress and fear:

If you are a sex worker, and you have a family and now you are on lockdown and there’s no money, the usual is social services kick in. Where is your food, where is your rental, where is your medical? […] How are you going to conduct your business? If I need $10 to pay for something, I would turn creative to think how I can get my money and how I can get transact on that work […] You work with fear and you try and earn some money. (Policymaker, Government-related entity)

### Lack of Access to COVID-19 Relief

Stakeholders highlighted how sex workers, like many others in society, have been impacted by the loss of work due to the COVID-19 pandemic. While there were opportunities to switch to other sources of income or rely on government relief for other sectors, sex workers were disadvantaged due to several factors. Firstly, stakeholders interviewed described how sex workers and especially those who identify as transgender women faced considerable stigma in society, and had experienced barriers to obtaining other jobs during this time:

We don’t know what our plan B might be. Because we had been in this work, the sex industry for so many years and we can’t think of any other line to turn to really. Which employer and realistically speaking, which potential employer would employ us without any related or prior experience? No work experience in another field, another line. During our chequered history, the social stigma of being a sex worker; all these has to be taken into consideration. Plus the social stigma of being a transgender in Singapore society. (Peer volunteer, CBO)

Stakeholders also stated that many sex workers were left out of government-funded COVID-19 relief. While a temporary relief fund was disbursed to all Singapore citizens and permanent residents who had lost their jobs or faced a loss of income at the start of the pandemic, our surveys revealed that only 56.2% (*n* = 77) of Singaporean sex workers surveyed had successfully applied for the initial temporary relief, which was provided on a per-application basis. Additionally, due to the illegality of sex work in Singapore and stringent requirements for documentation for work status or salary, longer-term relief was more difficult to obtain for some of our stakeholders:

You know the government just gave me $500 and the food is given by [CBO]. Everyday they send us food, that is the food we eat. […] But now we want to apply for the $800 [longer-term relief disbursement by the government]. They ask so many questions. Today I went down with [my sex worker colleague] so she can get $800. So terrible, this [government body]. They ask so many questions. You must have this document attached. You must have a salary, payslip attached. Oh my God. So now she is also suffering, her pocket no money, what do we do now? (Manager, Commercial entity)

With loss of income and lack of government relief, stakeholders expressed no surprise that sex work continued amidst the pandemic, despite the risks involved in doing so, whether owing to contact tracing measures and penalties or disease-related risks:

It makes sex workers afraid to go out and meet people. But they have to. They need to because they need to survive but they are afraid of getting the disease. Their mental health is not as good right now because they are always afraid of something that is going to happen; will they be caught by the authorities? Are they going to contract the disease? It is not just COVID that they are afraid right now. We have AIDS and all these diseases…For me, for the girls that I know, we are actually also afraid of contact tracing because we don’t want to get fined. If we do house calls, they trace that we are soliciting in our own house, obviously you get in trouble for that. (Peer volunteer, CBO)

### Unequal Impact of COVID-19 Among Sex Workers

Our quantitative findings revealed nuances in how COVID-19 had impacted various aspects of health and social conditions for sex workers in Singapore. Table 4 summarizes the linear regression models for the factors associated with a relative decrease in one’s income as a result of COVID-19, among the sex workers surveyed. Bivariable analyses revealed that being a transgender female (*C* = 8.10, *p* < .01) and non-Singaporean citizen or permanent resident (*C* = 14.51, *p* < .05) were positively associated with a greater loss of income. Multivariable analyses, adjusting for key demographic variables, indicated that being a transgender female (aC = 9.81, *p* < .01) was associated with a greater loss of income.

**Table 4:** Multivariable linear regression for relative percentage decrease in income

	Relative decrease in income (SGD %)						
	*β*	95% CI	*p*	Adj. *β*		95% CI	*p*
Age	−0.19	(−0.65, 0.26)	.406	−0.09		(−0.56, 0.38)	.699
Transgender female (ref = Cisgender female)	8.10**	**(2.75, 13.45)**	**.003**	9.81**		**(3.75, 15.87)**	**.002**
Stays with others or at work (ref = Stays or rents own home)	−1.36	(−12.84, 10.12)	.816	−2.68		(−14.25, 8.9)	.648
Venue-based sex work (ref = online or street based)	−1.26	(−13.08, 10.55)	.833	8.98		(−5.00, 22.95)	.206
Non-Singaporean (ref = Singapore citizen or permanent resident)	14.51*	**(1.86, 27.16)**	**.025**	11.13		(-2.09, 24.35)	.098

Adj, Adjusted; *β*, Unstandardized Coefficient; SGD, Singapore Dollars; CI, Confidence Interval

Statistically significant (*p* < .05) are highlighted in bold font; *p < .05; **p < .01; ***p < .001

Table 5 summarizes the Poisson regression models for the factors associated with increased food insecurity, housing insecurity, sexual compromise, as well as decreased access to medical and healthcare services. Multivariable analyses revealed that being a transgender female was positively associated with increased food insecurity (aPR = 1.23, 95% CI [1.08, 1.41]), housing insecurity (aPR = 1.28, 95% CI [1.03, 1.60]) and decreased access to medical and healthcare services (aPR = 1.74, 95% CI [1.23, 2.46]), being a venue-based sex worker was positively associated with increased food insecurity (aPR = 1.46, 95% CI [1.00, 2.13]), and being a non-Singaporean citizen or permanent resident was positively associated with increased housing insecurity (aPR = 2.59, 95% CI [1.73, 3.85]).

**Table 5:** Multivariable Poisson regression models for changes in health and social conditions among sex workers

	Increase in food insecurity		Increase in housing insecurity		Increase in sexual compromise		Decrease in access to medical care	
	aPR	95% CI	aPR	95% CI	aPR	95% CI	aPR	95% CI
Age	1.00	(0.99, 1.01)	0.99	(0.97, 1.01)	0.96	(0.92, 1.00)	0.98	(0.95, 1.01)
Transgender female (ref = Cisgender female)	1.23**	**(1.08, 1.41)**	1.28*	**(1.03, 1.60)**	1.08	(0.52, 2.26)	1.74**	**(1.23, 2.46)**
Stays with others or at work (ref = stays or rents own home)	0.79	(0.57, 1.10)	0.65	(0.37, 1.14)	2.05	(0.86, 4.89)	0.49	(0.19, 1.24)
Venue-based sex work (ref = online or street based)	1.46*	**(1.00, 2.13)**	1.21	(0.68, 2.15)	0.59	(0.16, 2.18)	1.13	(0.53, 2.39)
Non-Singaporean (ref = Singapore citizen or permanent resident)	0.95	(0.71, 1.29)	2.59***	**(1.73, 3.85)**	0.69	(0.15, 3.09)	1.55	(0.85, 2.80)

aPR, adjusted prevalence ratio; CI, confidence interval

Statistically significant (*p* < .05) are highlighted in bold font; *p < .05; **p < .01; ***p < .001

## Discussion

Our findings highlight how the COVID-19 pandemic has impacted Singapore’s sex work industry through the closure of venues for client access, as well as the accompanying movement control measures and fears around COVID-19 acquisition. Such loss of work and income for sex workers has led to an increase in food insecurity, housing insecurity, sexual compromise, as well as a decrease in access to medical and healthcare services among sex workers in Singapore. We also found that this impact was more keenly felt by some groups, such as transgender female, non-Singaporean and venue-based sex workers.

Our main finding that the pandemic has led to a loss of income among sex workers is not surprising and has been seen across the world (United Voices of the World, 2020). With loss of income accompanied by a lack of access to government relief, and deteriorating health and social conditions, it is unsurprising that sex workers have continued to work amidst the pandemic, exposing them to further risks of sexual compromise, sexually transmitted infections, and COVID-19. Many scholars and policymakers had made calls to action earlier on in the pandemic to highlight these exacerbating inequities (Adebisi et al., 2020; Lam, 2020a, b, c; Platt et al., 2020), and more recent studies across the world have validated these concerns—that sex workers are facing poor access to health services, and need to continue working to survive (Callander et al., 2020; Gichuna et al., 2020; Singer, Crooks, Johnson, Lutnick, & Matthews, 2020).

A key strength of this sequential exploratory mixed methods design was the utilization of insights from the stakeholder interviews for the development of the survey instrument in the subsequent quantitative phase of the study. This allowed the team to field a concise questionnaire while generating deeper insight into the nuances of how the COVID-19 pandemic has impacted sex workers in Singapore. Through this approach, we were able to quantify the loss of income among sex workers, as well as ascertain levels of food and housing insecurity, increase in levels of sexual compromise, as well as decreased access to medical and healthcare services.

While sex workers, in general, have been significantly affected by the COVID-19 pandemic, our study highlights certain subgroups of sex workers who have been more adversely impacted. Overall, we found that identifying as transgender women was associated with poorer outcomes, which comports with the evidence on how transgender women in general experience stigma across various settings and life situations (Operario, Yang, Reisner, Iwamoto, & Nemoto, 2014; White Hughto, Reisner, & Pachankis, 2015). Specifically, we found that being a transgender female sex worker was positively associated with a greater loss of income and risk of increased food and housing insecurity, as compared to being a cisgender female sex worker. These corroborated the insight generated from our stakeholder interviews, which suggested that transgender female sex workers were less likely to be able to find alternative jobs due to stigma. Specifically, such stigma is rooted in negative societal perceptions toward transgender women as well as sex workers in Singapore (Teo et al., 2019). This is consistent with findings elsewhere, which place transgender individuals at a disadvantage in accessing employment across the world and in Singapore (Granberg, Andersson, & Ahmed, 2020; Winter et al., 2018). Transgender female sex workers were also more likely than cisgender female sex workers to face decreased access to medical and healthcare services, which is unsurprising given how transgender women have historically faced considerable stigma in healthcare settings across the world (Kosenko, Rintamaki, Raney & Maness, 2013; White Hughto et al., 2015). Intersectionality provides an analytical lens to show how multiple interlocking identities, such as being a sex worker and transgender, reflect broader interconnected inequalities at the structural level (Crenshaw, 1991). Our findings reflect how such identities do not operate independently of one another, but are shaped in relation to broader social inequalities (Collins, 2015).

Among all outcomes measured in the survey, sexual compromise had the lowest magnitude of increase as a result of the COVID-19 pandemic, as well as the lowest baseline levels. Nevertheless, the presence of such conditions is consistent with experiences of sex workers in the region (Decker et al., 2010; Draughon Moret et al., 2016), as well as those around the world (Deering et al., 2014). While the results of multivariable analyses show that staying with others or at work was positively associated, and that being a venue-based or non-Singaporean citizen or permanent resident sex worker was negatively associated with an increase in sexual compromise, these findings were not statistically significant. Further studies are warranted to investigate the nature of sexual compromise that sex workers face in Singapore and the factors that exacerbate or drive such situations.

We are mindful of several limitations in this study. First, there may have been a selection bias in our sampling, in that our sample of sex workers surveyed may not have been representative of all sex workers’ experiences in Singapore. This may be due to the fact that many foreign sex workers had already left the country at the point of the survey and that our CBO partner’s reach may be limited to sex workers who are venue based. Nevertheless, our purposive sampling approach allowed us to ensure that we obtained enough respondents who operated outside of such venues as well, such as online or street-based sex workers. Second, a combination of recall and representativeness bias may have impacted the results of our findings when asking sex workers to compare their conditions in the year before COVID-19 and at present, as sex workers are more likely to be able to articulate a worsening of the situation given their present circumstances. Notwithstanding this limitation, our survey captures the existing realities and worsening situations of sex workers in Singapore as a result of COVID-19. Future research should explore sex workers’ experiences with the relaxation of COVID-19 control measures, providing greater perspective about the unique challenges faced by this key vulnerable population. Third, there was a lack of established or validated scales used in our survey questionnaire to measure our outcome variables, and further research is warranted to test the validity and reliability of such questions in future studies. Fourth, due to the need to keep the survey concise for purposes of field surveys, other outcomes such as mental health indicators and other potential confounders may have been left unmeasured. Finally, scholars have highlighted how community-based participatory research holds promise in reducing health disparities and power structures in research (Wallerstein & Duran, 2006); while the present study involves community members in its design, management, and execution, and situates itself toward a more community-driven approach along the continuum of community engagement in research (Key et al., 2019), further work can be done to create more democratic research structures in the sex work community.

The COVID-19 pandemic also brought about several changes to conventional methodological approaches to a mixed methods study, such as the conducting of qualitative interviews over teleconferencing software as well as the need to conduct such research rapidly to inform policies. We have previously reported some lessons learnt from the qualitative phase of this study in a separate research letter (Tan et al., 2020). As a potential source of bias, our qualitative interviews with stakeholders were conducted over teleconferencing software and may have affected the level of rapport that our interviewers could have built with participants. Nevertheless, we believe the impact of this bias would be minimal given that the topics focused on the broader impact of COVID-19 on sex workers, and did not focus on sensitive or personal information.

Public debate around the impact of COVID-19 on sex workers has largely been absent in Singapore, owing to the hidden and stigmatized nature of sex work; this has also been seen around and the world and have spurred calls to action by researchers, advocates, and members of the sex work community (Adebisi et al., 2020; Bromfield, Panichelli & Capous-Desyllas, 2021; Global Network of Sex Work Projects, 2020b; Lam, 2020a, b, c; Platt et al., 2020; UNAIDS, 2020b). This study therefore attempts to highlight and provide empiric evidence for the worsening conditions that sex workers face. In Singapore, past media coverage of sex workers during the COVID-19 pandemic have focused largely on cases that involve the breach of movement control measures, thus reproducing stigma toward sex workers rather than highlighting the situation that they are facing during this time (Lam, 2020a, b).

We conclude with several suggestions for policymakers in Singapore. Several national governments have implemented policies to provide relief to sex workers that include temporary legal status as well as stimuli packages for sex workers. For example, the Portuguese government granted temporary residency rights to all immigrants and asylum seekers who applied for residency in the country before 18 March 2020, when the state of emergency for COVID-19 was announced. These rights gave immigrants and asylum seekers access to social and health benefits, including access to the national health service, bank accounts and work and rental contracts. Governments have also helped sex workers look for new jobs, in addition to providing or enforcing free rent, such as in Groningen, Netherlands (Pieters, 2020; UNAIDS, 2020c). Furthermore, governments may consider waiving the stringent requirements for documentation and paperwork to becoming eligible for COVID-19 relief or for future emergencies. Considerations to undertake such interventions should be considered in Singapore as well to recognize sex work as an official form of work.

This study also points out the disproportionate impact of the pandemic on transgender sex workers due to years of institutionalized stigma and discrimination, and we recommend that policymakers set up a taskforce to address such barriers to achieving health equity for transgender individuals. Similarly, this study points out the potential vulnerability of non-Singapore citizens and permanent residents to the impact of the pandemic, and we recommend that strategies strengthen aspects of local employment legislation to protect such work pass holders be considered and implemented. Finally, additional funding or redirection of resources toward CBOs such as Project X would be instrumental in mitigating the negative impact of COVID-19 on sex workers, as these organizations play a vital role in bridging resources for sex workers in need during the COVID-19 pandemic.

## Supplementary Information

Below is the link to the electronic supplementary material.

Supplementary file1 (DOCX 19 kb)

Supplementary file2 (DOCX 21 kb)

Supplementary file3 (DOCX 39 kb)

## Data Availability

The data that support the findings of this study are available from the corresponding author, Rayner Kay Jin Tan, upon reasonable request.
