# Supplementary material for: The Impact of the Coronavirus Disease (COVID-19) on the Health and Social Needs of Sex Workers in Singapore
Source: Arch Sex Behav. 2021 Jun 30;50(5):2017–29. doi: 10.1007/s10508-021-01951-8 (PMC8244454; doi:10.1007/s10508-021-01951-8)
Supplement: Supplementary file 1 — Supplementary file1 (DOCX 19 kb) [file 10508_2021_1951_MOESM1_ESM.docx]

**Semi-Structured Interview Topic Guide**

| **Theme** | **Sample Questions / Prompts** |
| --- | --- |
| Demographics | 1. Organization or community group that individual is representing 2. Role in organization (e.g. volunteer, 3. Past experience working with the sex work community 4. Professional expertise |
| Organization / Mapping of resources and flow of information  (If relevant) | 1. Vision and mission of organization 2. History and origin of organization 3. Key activities and operations of organization 4. Main beneficiaries of organization 5. Main funding sources for organization 6. Organization’s relationship with other community groups or organizations (Flow of resources and information) |
| COVID-19’s organizational impact | 1. How have your organization and its operations been afected by COVID-19? (Start from January 2020 and subsequent months as new measures were implemented) 2. Has staffing been affected? If yes or no, what are the reasons? 3. Has funding been affected? If yes or no, what are the reasons? 4. How has it affected ongoing grants and programmes? 5. How has your organization pivoted to cope with the changes that COVID-19 and the concomitant measures by the government have brought on? |
| COVID-19’s impact on sex work | (Capture what it was like before, and how it is like now due to COVID-19)   1. What changes have you seen on the ground as a result of COVID-19? 2. What changes have you seen since the frist imported case from China was confirmed on 23 January? 3. What changes have you seen in sex work trends as a result of the closure of entertainment establishments? 4. What changes have you seen in sex work trends as a result of the circuit breaker measures? |
| COVID-19’s impact on sex workers | 1. Would you say that sex workers in Singapore are a heterogenous group? How so? 2. How have these different groups of sex workers been affected by COVID-19? 3. To your knowledge, do you know any sex workers who have continued working in spite of the closure of entertainment establishments or the circuit breaker measures? Why do you think this is so? 4. How has COVID-19 affected the livelihood of sex workers? 5. In what ways can sex workers overcome such changes to their own jobs and personal lives? Do you know whther they have switched to other jobs What jobs? 6. Have any policies or financial aids been helpful for sex workers? How so? 7. Is there a need for reskilling of sex wokers for other work? |
| COVID-19’s impact on clients of sex workers | 1. Who do you think are typical clients of sex workers in Singapore? Are there differences in such clientele across the different groups of sex workers? 2. How do you think COVID-19 has affected this dynamic and the clientele / demand for sex work in Singapore? 3. In what ways have business practices changed to meet the demands of clients in times of COVID-19? |
| Recommendations | 1. What do you think needs to be done moving forward to address the needs of sex workers in Singapore in times of COVID-19? (Prompt: Financial, psychological, social and physical needs) |
