# Supplementary material for: The Impact of the Coronavirus Disease (COVID-19) on the Health and Social Needs of Sex Workers in Singapore
Source: Arch Sex Behav. 2021 Jun 30;50(5):2017–29. doi: 10.1007/s10508-021-01951-8 (PMC8244454; doi:10.1007/s10508-021-01951-8)
Supplement: Supplementary file 2 — Supplementary file2 (DOCX 21 kb) [file 10508_2021_1951_MOESM2_ESM.docx]

**Coding Frame**

1. **Social change as a result of COVID-19**
   1. Control measures implemented
   2. Impact on community-based organizations
   3. Impact on sex workers
2. **Institutional factors that mediate experience of COVID-19**
   1. Existing government structures
   2. Existing NGO structures
3. **Individual factors that mediate experience of COVID-19**
   1. Demographic factors
   2. HIV status
   3. Existing housing situation
   4. Existing socioeconomic Status
   5. Ability to navigate technology
   6. Nature of sex work
      1. No paperwork for sex workers’ salary
      2. Not able to pursue protective measures
   7. Perceptions of COVID-19
   8. Social factors
      1. HIV criminalization
      2. Illegality of sex work
      3. Lack of government and police support
      4. Lack of skills in other areas
      5. Stigma towards being transgender
      6. Stigma towards sex workers
      7. Vulnerability of sex workers
4. **Additional demands as a result of COVID-19**
   1. Macro demands
      1. Lack of jobs in the market
   2. Micro demands
      1. Food security
      2. Housing security
      3. Job security
      4. Lack of access to medical services
      5. Lack of money
      6. Medical expenses
      7. Mental and emotional security
      8. Physical security
      9. Power differential with clients
      10. Supporting family back home
5. **Resources available to sex workers**
   1. Food from NGOs
   2. Government relief
   3. Help from employer
   4. Medication access
   5. Monetary support from NGOs
   6. NGO Relief Vouchers
   7. NGO rental negotiations
   8. Personal protective supplies from NGO
6. **Adjustments in response to COVID-19**
   1. Carry on sex work
   2. Do nothing for now
   3. Finding ad-hoc / part-time work
7. **Recommendations**
   1. Emergency hotline
   2. Enhanced surveillance
   3. Getting clients used to new normal
   4. Government support (recognition, expand criteria of self-employment)
   5. Help centre for sex workers (NGO)
   6. Monetary support
   7. More police support
   8. Personal protective measures
   9. Reducing stigma
   10. Reskilling for those who want it
