# Supplementary material for: The Impact of the Coronavirus Disease (COVID-19) on the Health and Social Needs of Sex Workers in Singapore
Source: Arch Sex Behav. 2021 Jun 30;50(5):2017–29. doi: 10.1007/s10508-021-01951-8 (PMC8244454; doi:10.1007/s10508-021-01951-8)
Supplement: Supplementary file 3 — Supplementary file3 (DOCX 39 kb) [file 10508_2021_1951_MOESM3_ESM.docx]

**Impact of COVID-19 on the sex work industry in Singapore**

**Version 4: 23 May 2020**

| **#** | **Question** | **Response categories** | |
| --- | --- | --- | --- |
|  | What is your age? | [Open-ended response] | |
|  | What is your gender? | ☐ Cisgender Female  ☐ Transgender Female  ☐ Cisgender Male  ☐ Transgender Male  ☐ Others: ___________________ | |
|  | What is your residency status? | Singapore citizen  Singapore permanent resident  Others: ___________________ | |
|  | What is your country of birth? | [Open-ended response] | |
|  | What visa do you currently hold in Singapore? | Social visit pass  Dependent pass  Long term visit pass / long term visit pass +  Work permit  S pass  Employment pass  Special pass  Others: ___________________ | |
|  | How long have you been working in Singapore? | < 1 month  About 1 month  About 2 months  About 3 months  3 to 6 months  6 to 12 months  More than 12 months | |
|  | [If SVP selected in Q4] In one year, how many times do you typically come to Singapore to work? | [Open-ended response] | |
|  | [If SVP selected in Q4] On average, how long do you stay in Singapore during each visit? | About 1 week  About 2 weeks  About 3 weeks  About 1 month  Between 1 to 2 months  About 2 months  Between 2 to 3 months  About 3 months  > 3 months | |
|  | Where do you currently stay? | I rented a bed/room/flat on my own  I stay with friends or family members without having to pay  I stay in a brothel  Others: ___________________ | |
|  | Where does sex work take place for you? You may select more than one option. (you can tick more than 1 box) | Legal licensed brothels  Unlicensed brothels  Entertainment establishments, bars, clubs  I get my clients from the streets (street-walker)  I get clients online via websites  I get clients through mobile apps  Hotels / short-time rooms  Others: ____________________ | |
|  | Do you have another paid job? | No  Yes: please specify _________ | |
| **Brothel-Based Sex Worker Questions** | | | |
|  | If licensed brothel-based: Which area(s) do you typically work at? You may select more than one option. | Geylang area  Little India area  Others: ___________________ | |
|  | (If licensed brothel was selected) How many days in a week do you work at the brothel? | 1 day  2 days  3 days  4 days  5 days  6 days  7 days  Others: ___________________ | |
|  | How many clients do you have on a typical work day at the brothel? (Before COVID-19 started in Singapore on 23^rd^ January 2020) | [Open-ended response] | |
|  | What proportion of these clients are regular or repeat clients? | Less than 10%  10%  20%  30%  40%  50%  60%  70%  80%  90%  Almost all of them are regulars or repeat clients | |
|  | On average, what proportion of your clients at the brothel are Singaporeans? | Less than 10%  10%  20%  30%  40%  50%  60%  70%  80%  90%  Almost all of them are Singaporean | |
|  | For your non-Singaporean clients at the brothels, which countries do your clients typically come from?  *(List from MOM’s source countries for work permit holders)*  *You can tick more than 1 box* | Malaysia  People’s Republic of China  India  Bangladesh  Indonesia  Sri Lanka  Thailand  Myanmar  Phillippines  Hong Kong  Macau  South Korea  Taiwan  Others: ___________________ | |
| **Non-Brothel Based Sex Worker Questions** | | | |
|  | (If non-brothel-based was selected) How many days in a week do you work outside of the brothel? | 1 day  2 days  3 days  4 days  5 days  6 days  7 days  Others: ___________________ | |
|  | Where do you typically meet your clients? | I book a hotel room  Private home eg HDB flat, maisonette owned by pimp/agent  Shop owned by agent or pimp  Client books a hotel room  Client’s house  My own residence  In public areas eg street, park  In entertainment establishments (e.g. bar, club, karaoke lounge)  Others: ___________________ | |
|  | How many clients do you have on a typical work day outside of the brothel? (Before COVID-19 started in Singapore on 23^rd^ January 2020) | [Open-ended response] | |
|  | What proportion of these clients are regular or repeat clients? | Less than 10%  10%  20%  30%  40%  50%  60%  70%  80%  90%  Almost all of them are regulars or repeat clients | |
|  | On average, what proportion of your non-brothel-based clients are Singaporeans? | Less than 10%  10%  20%  30%  40%  50%  60%  70%  80%  90%  Almost all of them are Singaporean | |
|  | For your non-Singaporean clients outside of the brothels, which countries do your clients typically come from?  *(List from MOM’s source countries for work permit holders)*  *You can tick more than 1 box* | Malaysia  People’s Republic of China  India  Bangladesh  Indonesia  Sri Lanka  Thailand  Myanmar  Phillippines  Hong Kong  Macau  South Korea  Taiwan  Others: ___________________ | |
| **More About Clients** | | | |
|  | For your Singaporean clients, how would you describe their type of profession? | White collar professionals  Blue collar workers  Others: ___________________ | |
|  | For your non-Singaporean clients, how would you describe their type of profession? | Expatriate (White collar etc.)  Business travelers  Low-wage foreign workers  Tourists  Others: ___________________ | |
|  | [If low wage foreign worker was selected] Do you happen to know where they stay? | [Open-Ended] | |
|  | Do you spend additional time with your clients when not having sex with them? For example, having a meal, watching a movie etc. | I never spend time with my clients after having sex with them  Rarely  Sometimes  About half the time  Most of the time  Almost all the time  All the time | |
|  | Where do you typically go with your clients when you spend time together with them? You may select more than one option. | Have a meal at a nice restaurant  Have a meal nearby where we will have/had sex  Head out shopping  Head out for a movie  Head out for a stroll or walk at the beach or a park  Others: ___________________ | |
| **COVID-19’s Impact of Sex Work** | | | |
|  | What precautions did your brothel take to protect you and your clients from the coronavirus before they were ordered to close on 26 March 2020? | Safe distancing measures (e.g. reduce number of workers, reduce number of rooms or beds used)  Reduced the number of customers per day  Improving cleanliness of premises (e.g. cleaning more often)  Temperature screening of employees  Temperature screening of clients  Travel declaration forms for employees  Travel declaration forms for clients  Providing protective equipment at the brothel (e.g. masks and hand sanitizers)  Others: ___________________ | |
|  | Here are some precautions that some people have taken to protect themselves from the coronavirus. Did you practice any of these throughout the course of your work (both inside and outside of brothels) before the circuit breaker came into effect on 7 April 2020?? | Answer yes, no or prefer not to say for each of the following:  **I made efforts to wash and sanitize my hands more often**  **I made efforts to wear a mask more often**  **I conducted temperature screening for myself**  **I conducted temperature screening for clients**  **I asked my clients about their travel histories**  **I asked my clients about their symptoms**  **I looked out for symptoms in my clients**  **I reduced the number of clients that I had**  Did you do anything else as a precaution?: ___________________  Not applicable – I stopped sex work | |
|  | [Brothel-based workers] Should your brothel be allowed to reopen, would you be keen on resuming sex work immediately? | Yes  No  Don’t know | |
|  | [Non-brothel-based workers] Should it be allowed, would you be keen on resuming sex work immediately? | Yes  No  Don’t know | |
|  | Should you be allowed to do sex work now, What are some precautions that you will take to protect yourself? | [Open-ended] | |
| **COVID-19’s Impact on Sex Worker’s Well-Being** | | | |
|  | What do you consider to be your own probability of getting COVID-19? | Extremely unlikely (7 point scale)  Extremely likely | |
|  | How severe would getting COVID-19 be for you (how seriously ill do you think you will be)? | Not severe (7 point scale)  Very severe | |
|  | I know how to protect myself from coronavirus | Not at all (7 point scale)  Very much so | |
|  | Was there a time when you were not able to pay rent on time? | **In the year before COVID-19:**  Yes  No  Don’t know | **As a result of COVID-19:**  Yes  No  Don’t know |
|  | In terms of where you stay, have you had to move from two times or more within a month? | **In the year before COVID-19:**  Yes  No  Don’t know | **As a result of COVID-19:**  Yes  No  Don’t know |
|  | I worried whether my food would run out before I for money to buy more. | **In the year before COVID-19:**  Often true  Sometimes true  Never true  Don’t know | **As a result of COVID-19:**  Often true  Sometimes true  Never true  Don’t know |
|  | The food I bought just didn’t last and I didn’t have money to get more. | **In the year before COVID-19:**  Often true  Sometimes true  Never true  Don’t know | **As a result of COVID-19:**  Often true  Sometimes true  Never true  Don’t know |
|  | I had to compromise on my own usual sexual health practices with my clients. | **In the year before COVID-19:**  Often true  Sometimes true  Never true  Don’t know | **As a result of COVID-19:**  Often true  Sometimes true  Never true  Don’t know |
|  | I was able to access medical or healthcare services that I needed. | **In the year before COVID-19:**  Often true  Sometimes true  Never true  Don’t know | **As a result of COVID-19:**  Often true  Sometimes true  Never true  Don’t know |
|  | How much do you usually pay for rent for yourself on a monthly basis? | [Open-ended response] | |
|  | How much do you usually spend on food for yourself on a monthly basis? | [Open-ended response] | |
|  | How much do you usually spend on medication for yourself on a monthly basis? | [Open-ended response] | |
|  | How much do you usually spend on your partner or family members (e.g. sending money home, paying for other family members’ food etc.) | [Open-ended response] | |
|  | How much do you typically earn (in SGD) in a regular month before the circuit breaker measures? | [Open-ended response] | |
|  | How much do you earn (in SGD) now due to COVID-19? | [Open-ended response] | |
|  | If you receive money regularly from other sources, how much money (in SGD) have you received on average on a monthly basis? | [Open-ended response] | |
|  | [Singaporean citizens and PR only] Were you aware of the Temporary Relief Fund for one-time $500.00 assistance that was launched in April 2020? | Yes  No  Don’t know | |
|  | [Singaporean citizens and PR only] If you were aware, were you eligible to apply for the one-off $500.00 assistance? | Yes, I was eligible and successfully applied for it  Yes, I was eligible but did not apply for it  No, I was not eligible for it | |
|  | [Eligible but did not apply for it] Why did you not apply for the fund? **You may select more than one option** | I didn’t know how to apply for it  I didn’t have a bank account  I didn’t have an internet connection  I didn’t have Singpass  I did not feel comfortable applying for the fund in-person (i.e. at social service office or community centre)  I had a negative experience while trying to apply for the fund in-person  I was not able to provide proof of loss of job or income  I only heard about the fund after the application period was over  Others: ____________________ | |
|  | [Not eligible to apply for it it] Why were you not eligible for the fund? | I was not able to provide proof of loss of job or income  I did not experience any loss of job or income of more than 30%  My gross monthly household income exceeded $10,000 or income per capita exceeded $3,100.  I am currently on Comcare assistance  Others: ____________________ | |
|  | [Foreign work pass holders only] Were you aware of the foreign worker levy rebates of $750.00 that your employer received from the government? | Yes  No  Don’t know | |
|  | [If yes to previous question] Did your employer pass on the rebate to you in any way? | Yes, my employer gave me the full rebate and I did not have to work  Yes, my employer gave me the full rebate and I had to work for it  Yes, my employer gave me part of the rebate and I did not have to work  Yes, my employer gave me part of the rebate and I had to work for it.  No, I did not receive any rebate or allowance from my employer | |
|  | Have you tried to apply for any other jobs in the last 3 months? | Yes, I successfully applied for a full-time job  Yes, I successfully applied for a part-time job  Yes, but I wasn’t successful in getting any jobs  No  Prefer not to say | |
|  | [If yes and successful] May I ask what job role you successfully applied for? | [Open-ended response] | |
|  | [If yes but not successful] Were you aware about why you weren’t successful in your job application? | [Open-ended response] | |
|  | [If no] Would you consider applying for a part-time job if it was made available to you? Please explain why so [if yes, or no]. | Yes  No  Don’t know  Explanation why: ________________________________ | |
|  | If I developed respiratory symptoms (e.g. runny nose, cough) today, I would | Go to a polyclinic  Go to a general practitioner’s clinic  Go to a hospital’s A&E department  Go to NCID  Go to a Traditional Chinese Medicine provider  Go to a friend/pimp/brothel owner?  Stay at home  Do none of the above | |
|  | [If picked 1-4] I trust that the healthcare provider will manage my illness appropriately | Strongly agree  Agree  Disagree  Strongly disagree | |
